# The Long-Term Uptake of Home Spirometry in Regular Cystic Fibrosis Care: Retrospective Multicenter Observational Study

**DOI:** 10.2196/60689

**Published:** 2025-01-09

**Authors:** Pia Bertram, Martinus C Oppelaar, Michiel AGE Bannier, Monique HE Reijers, Hester van der Vaart, Renske van der Meer, Josje Altenburg, Lennart Conemans, Bart L Rottier, Marianne Nuijsink, Lara S van den Wijngaart, Peter JFM Merkus, Jolt Roukema

**Affiliations:** 1 Department of Pediatric Pulmonology Amalia Children’s Hospital Radboud University Medical Center Nijmegen Netherlands; 2 Department of Pediatric Pulmonology MosaKids Children’s Hospital Maastricht University Medical Centre+ Maastricht Netherlands; 3 Radboud University Medical Center Department of Pulmonary Diseases Nijmegen Netherlands; 4 Department of Pulmonary Diseases University Medical Center Groningen University of Groningen Groningen Netherlands; 5 Department of Pulmonology Haga Teaching Hospital The Hague Netherlands; 6 Department of Respiratory Medicine Amsterdam University Medical Centers University of Amsterdam Amsterdam Netherlands; 7 Department of Respiratory Medicine Maastricht University Medical Centre+ Maastricht Netherlands; 8 Division of Respiratory & Age-related Health Department of Respiratory Medicine NUTRIM Institute of Nutrition and Translational Research in Metabolism Maastricht Netherlands; 9 Department of Pediatric Pulmonology and Pediatric Allergology Beatrix Children's Hospital University Medical Center Groningen Groningen Netherlands; 10 Groningen Research Institute for Asthma and COPD University Medical Center Groningen University of Groningen Groningen Netherlands; 11 Juliana Children’s Hospital Haga Teaching Hospital The Hague Netherlands

**Keywords:** telemonitoring, eHealth, spirometry, adherence, pulmonary medicine, home spirometers, cystic fibrosis, autosomal disease, treatment, remote monitoring, survival analyses, frequency, digital health, telehealth

## Abstract

**Background:**

Home spirometers have been widely implemented in the treatment of people with cystic fibrosis (CF). Frequent spirometry measurements at home could lead to earlier detection of exacerbations. However, previous research indicates that the long-term use of home spirometry is not well maintained by people with CF.

**Objective:**

We aimed to gain insight into the long-term uptake of home spirometry in regular multicenter CF care.

**Methods:**

Home spirometers combined with a remote monitoring platform were introduced in the treatment of people with CF in 5 Dutch CF centers starting in April 2020. Usage data from April 2020 to December 2022 were analyzed retrospectively. Survival analyses were conducted to assess use consistency over time, and *t* tests were used to evaluate the impact of increased pulmonary symptoms on home spirometry frequency. The effect of the initiation of a new treatment, Elexacaftor/Tezacaftor/Ivacaftor, on use frequency over time was assessed in a subgroup of participants with repeated measures ANOVA.

**Results:**

During the observation period, a total of 604 people with CF were enrolled in the remote monitoring platform and 9930 home spirometry measurements were performed. After the initiation of home spirometry use, the number of users declined rapidly. One year after the initiation, 232 (54.2%) people with CF stopped using home spirometry. During the observation period, 67 (11.1%) users performed more than 20 measurements. Furthermore, the number of consistent home spirometry users decreased over time. After 600 days, only 1% of users had measured their lung function consistently every 31 days. Use frequency slightly increased during periods with increased pulmonary symptoms (ΔMean=0.45, *t*_497.278_=–4,197; *P*<.001) and showed an initial rise followed by a decrease after starting treatment with Elexacaftor/Tezacaftor/Ivacaftor (ΔMean=0.45, *t*_497.278_=–4,197; *P*<.001).

**Conclusions:**

Consistent uptake of home spirometry in people with CF is low but increases around periods of changing symptoms. A clear strategy for the organization of remote care seemed to improve the long-term uptake of home spirometry. Nevertheless, home spirometry and its intensity are not a goal on their own but should be used as a tool to reach individual goals within local contexts.

## Introduction

Cystic fibrosis (CF) is a recessive autosomal disease caused by dysfunction of the CF transmembrane conductance regulator (CFTR) channel. Dysfunction of the CFTR channel leads to increased pulmonary mucus viscosity that causes chronic inflammation and predisposes to recurrent infections [1]. Early detection and treatment of pulmonary decline is essential to prevent mortality and morbidity in people with CF. The forced expiratory volume in 1 second (FEV1) measured by spirometry is the most reliable clinical outcome for this end [2,3].

Portable home spirometers that measure FEV1 have been available for multiple years. However, they have recently become more accessible thanks to advances in digital patient environments, the widespread availability of smartphones to operate them with, and a pivot toward home monitoring driven by the COVID-19 pandemic [4,5]. In theory, home spirometers allow people with CF to identify lung function decline sooner which could help them seek timely medical intervention and thus reduce mortality, morbidity, and health care costs [6].

Early detection of pulmonary decline with home spirometry would require regular self-monitoring of people with CF over extended periods, also when their condition is stable. In recent work, we found there is little incentive for people with CF to frequently self-monitor during periods of well-being, or when there is no reduction in therapy burden elsewhere [7]. This is in line with findings from previous prospective studies that reported declines in monitoring adherence to study protocols over time, especially in adults [6,8-11].

Understanding how people with CF incorporate home spirometry into their treatment routine is crucial for evaluating its added value. There is a specific need for more insights into the long-term uptake of home spirometry in regular CF care. This study aimed to examine the uptake of home spirometry in regular CF care in 5 Dutch CF centers over 2.5 years. From previous work, we hypothesized that the uptake of home spirometry is not well-maintained over time, that home spirometry uptake increases during periods with increased pulmonary symptoms, and that home spirometry uptake declines after the initiation of treatment with a new drug that specifically targets the CFTR channel (Elexacaftor/Tezacaftor/Ivacaftor) [7].

## Methods

### Study Design

This was a retrospective, multicenter, observational study conducted in 5 Dutch CF centers (Radboud University Medical Center Nijmegen, University Medical Center Groningen, Maastricht University Medical Center, Amsterdam University Medical Center, Haga Hospital, The Hague).

### Remote Monitoring Platform

Home spirometry testing combined with a remote monitoring platform (RMP) was implemented on April 1, 2020, to prevent unnecessary outpatient visits during the COVID-19 pandemic in 5 Dutch CF centers. Details of the RMP have been published elsewhere [7]. In short, the RMP was used in addition to regular care in order to reduce physical hospital visits when possible. Considering the turbulent period of the COVID-19 pandemic, no formal implementation period or protocols were used. Instead, the implementation of the RMP and its use varied between departments based on their local settings, capacities, and needs. Therefore, RMP implementation across the 5 centers and enrollment of individual users occurred gradually during the COVID-19 period [7]. As a general rule, people with CF were asked to measure their lung function monthly, but health care professionals and users had the freedom to deviate from this based on individual contexts.

Home spirometry was performed with the Spirobank Smart (Medical Internet Research). Home spirometers were donated by the Dutch Cystic Fibrosis Foundation. In addition, 4 of the 5 CF centers used a digital symptom questionnaire based on the Modified Fuchs criteria to track symptoms indicative of a pulmonary exacerbation. How often this questionnaire was used varied between centers and people with CF. If 2 or more symptoms were reported, automated feedback provided the advice to measure the lung function and contact the CF team as needed [12].

### Study Population

This study included all people with CF who measured their lung function at home with the RMP. Home spirometry and symptom survey data of all users of the RMP were extracted anonymously. As this was a retrospective observational trial with anonymous data, no formal eligibility criteria were used. In practice, eligibility for enrollment on the RMP is decided by the treating health care professionals and patients themselves based on whether the user is able and willing to measure their lung function at home. In general, children had to be older than 6 years old to be considered for enrolment on the RMP. Individual start dates of Elexacaftor/Tezacaftor/Ivacaftor were available for a subgroup of people with CF who participated in a previous study and who gave informed consent for additional data collection [7].

### Primary and Secondary Outcome Measures

Primary outcomes included (1) the median number of days between each consecutive home spirometry measurement day during the observation period (time-to-next lung function [TTN]) and (2) the proportion of users who continued self-monitoring with consistent frequencies (ie, monthly, 2-monthly, or quarterly) over time. Secondary outcomes included (1) the difference in the average intra-person frequency of home spirometry measurements between periods of increased pulmonary symptoms (ie, a symptom survey score ≤5) and few pulmonary symptoms (ie, a symptom survey score ≥6) and (2) the difference in the average intra-person frequency of home spirometry following initiation of Elexacaftor/Tezacaftor/Ivacaftor.

### Statistical Analysis

All statistical analyses were conducted using IBM SPSS Statistics (version 27). User characteristics were described for all users and user groups based on home spirometry uptake categories (ie, 0, 1-20, and >20 unique measurement days). User characteristics of the group without measurements and the group with >20 unique measurement days were compared with each other using the Pearson chi-square test for proportions. To compare uptake categories with CF departments the Monte Carlo method was used to estimate the Fisher exact test. As data of users were gathered anonymously, only age, sex, and treating CF centers could be described. Treating CF centers were anonymized.

Consistent uptake of home spirometry was assessed using the Kaplan-Meier estimator. A total of 2 different definitions of consistent uptake were used in 2 separate analyses. First, we examined the number of users who self-monitored every 31, 62, or 93 days without breaking this pattern (ie, TTN remained below 31, 62, or 93 days). A break in this pattern was considered a loss of survival. Second, we allowed 1 break in the pattern after every 2 measurements following the pattern of 31 or 62 days. The break could be no longer than twice the number of the pattern (ie, 62 or 124 days, respectively). Users who had never measured their lung function or only once were excluded from the analyses. Separate subgroup analyses were performed for age categories at the first measurement, CF centers, and the adult and pediatric CF departments of the CF centers.

An independent *t* test was used to compare the mean number of home spirometry measurement days within 28 days of all symptom surveys with a score ≤5 (ie, a period of increased symptoms) with those taken within 28 days of all symptom surveys with a score ≥6 (ie, a period of few symptoms). In a sensitivity analysis, the intraindividual change in home spirometry use related to the number of symptoms was examined. A dependent *t* test was used to compare the number of home spirometry measurement days during the first period of increased symptoms of an individual after the initial 100 days of use with the closest period of few symptoms.

Changes in use frequency after the introduction of Elexacaftor/Tezacaftor/Ivacaftor were examined in a subgroup of people with CF. Data on Elexacaftor/Tezacaftor/Ivacaftor use was only available for a subgroup of 81 users who participated in a previous study [7]. Change in frequency of use was assessed with repeated measures ANOVA between multiple intervals around the initiation of Elexacaftor/Tezacaftor/Ivacaftor: the 30 days before initiation of Elexacaftor/Tezacaftor/Ivacaftor; and 0-30 days, 30-60 days, 60-90 days, and 90-120 days after initiation of Elexacaftor/Tezacaftor/Ivacaftor. Users were only included if they started Elexacaftor/Tezacaftor/Ivacaftor at least 120 days before the end of the study period.

### Ethical Considerations

Local ethical committees waived formal approval considering the negligible burden of participation and absence of imposed risks (file number local ethical committee Arnhem-Nijmegen region: 2021-13214). A subgroup of people with CF provided electronic informed consent for the use of additional pseudonymized data to answer the research questions of this study.

## Results

### Patient Characteristics

A total of 604 people with CF were included in the analysis. Demographics are presented in Table 1. The median time people with CF were enrolled on the RMP during the observation period was 816 (IQR 716-856, range 37-998) days. Throughout the 998-day observation period, a total of 9930 home spirometry measurements were performed of which 5104 were unique day measurements. Overall, 428/604 (70.1%) people with CF performed at least 1 home spirometry measurement, 365/604 (60.4%) had 2 or more unique home spirometry measurements days, and this number decreased further with more unique measurement days (Table 1). One year after the initiation of home spirometry, 232 (54.2%) people with CF stopped using home spirometry. For users with 2 or more unique home spirometry measurement days (N=365), the median participant TTN was 25 (IQR 12-76, range 1-541) days for the total period. The median number of days between the first and last unique home spirometry day was 334.5 (IQR 59.25-632.75, range 0-966).

**Table 1:** User characteristics of total population and subgroups based on the number of unique measurement days (none, 1-20, and >20).

		Total	No measurements	1-20 measurements	>20 measurements
		604 (100)	176 (29.1)	361 (59.8)	67 (11.1)
**Sex, n (%)**					
	Male	307 (50.8)	97 (55.1)	180 (49.9)	30 (44.8)
	Missing	34 (5.6)	9 (5.1)	21 (5.8)	4 (6)
**Age category^a,b^, n (%)**					
	<12 years	85 (14.1)	27 (15.3)	39 (10.8)	19 (28.4)
	12-18 years	75 (12.4)	24 (13.6)	37 (10.2)	14 (20.9)
	>18 years	432 (71.5)	123 (69.9)	277 (76.7)	18 (47.8)
	Missing	12 (2.0)	2 (1.1)	8 (2.2)	2 (3.0)
**CF^c^ department^d,e,f^, n (%)**					
	1	31 (5.1)	4 (2.3)	20 (5.5)	7 (10.4)
	2	49 (8.1)	7 (4.0)	37 (10.2)	5 (7.5)
	3	16 (2.6)	0 (0)	6 (1.7)	10 (14.9)
	4	46 (7.6)	11 (6.3)	23 (6.4)	12 (17.9)
	5	34 (5.6)	24 (13.6)	10 (2.8)	0 (0)
	6	136 (22.5)	59 (33.5)	75 (20.8)	2 (3.0)
	7	51 (8.4)	7 (4.0)	29 (8.0)	15 (22.4)
	8	76 (12.6)	11 (6.3)	60 (16.6)	5 (7.5)
	9	28 (4.6)	16 (9.1)	11 (3.0)	1 (1.5)
	10	125 (20.7)	35 (19.9)	82 (22.7)	8 (11.9)
	Missing	12 (2.0)	2 (1.1)	8 (2.2)	2 (3.0)
TTN^g^, median (IQR)		25 (12-76)	—^h^	34 (14-91)	13 (7-17)
Time in days between first and last measurement, median (IQR)		334.5 (59.25-632.75)	—	237 (21-524.5)	779 (633-832)

^a^Significant difference between the subgroup without measurements and the subgroup with >20 measurements based on the Pearson chi-square test of proportions.

^b^Significant difference between the subgroup with 1-20 measurements and the subgroup with >20 measurements based on the Pearson chi-square test of proportions.

^c^CF: cystic fibrosis.

^d^Significant difference between the subgroup without measurement and the subgroup with 1-20 measurements based on the Monte Carlo estimated Fisher exact test.

^e^Significant difference between the subgroup without measurements and the subgroup with >20 based on the Monte Carlo estimated Fisher exact test.

^f^Significant difference between the subgroup with 1-20 measurements and the subgroup with >20 measurements based on the Monte Carlo estimated Fisher exact test.

^g^TTN: time-to-next lung function measurement.

^h^Not applicable.

### Long-Term Use

**Figure 1:**
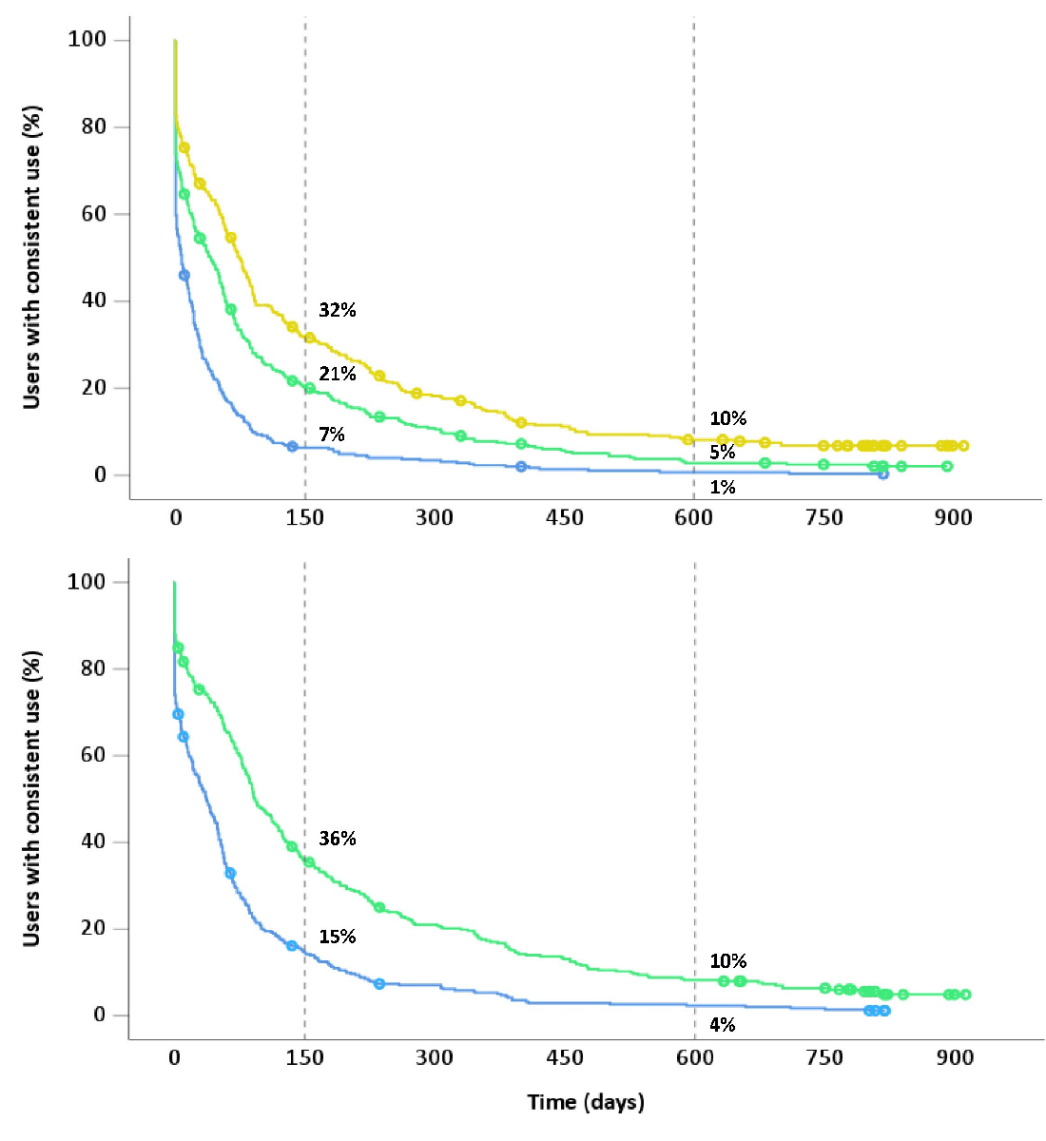
Kaplan-Meier survival curve of the consistency of home spirometry use (n=365). Upper panel: Consistent users with every FEV1 measurement performed with a maximum time-to-next lung function of 31, 62, or 93 days. Lower panel: consistent users with a more lenient approach, where one FEV1 measurement can be performed with a longer time-to-next lung function if two subsequent measurements are taken with a maximum time-to-next lung function of 31, or 62 days. People with cystic fibrosis who are consistent users at the end of the observation period are censored with dots. Yellow: 93-day interval; green: 62-day interval; blue: 31-day interval.

At 600 days, only 1% of users had measured their lung function at home consistently every 31 days. The decline was largest during the first 150 days across all intervals. For the more lenient criteria, the decline was less steep but followed a similar curve. Figure 1 shows Kaplan-Meier curves for the different uptake patterns. On average, consistent use frequency patterns were broken after 41 days (95% CI 31-51 days) for the 31-day interval, after 105 days (95% CI 86-123 days) for the 62-day interval, and after 172 days (95% CI 95-118 days) for the 92-day interval. After 150 days, 93% of users had broken the 31-day pattern, 79% had broken the 62-day pattern, and 68% had broken the 93-day pattern (Figure 1).

Multimedia Appendix 1 shows Kaplan-Meier curves for the strict use frequency patterns with subgroups based on age categories (6-12 years, 12 -18 years, ≥18 years; Figure S1 in Multimedia Appendix 1), sex (Figure S2 in Multimedia Appendix 1), treating CF center (Figure S3 in Multimedia Appendix 1), and age categories within treating CF center (<18 years within treating CF center vs ≥18 years within treating CF center; Figure S4 in Multimedia Appendix 1). There was no difference between long-term use consistency and sex. Children and teenagers generally seemed to use their home spirometer consistently longer than adults. Average consistent use frequency patterns were maintained longer in one CF center than in the others with a mean survival of the strict 31-day pattern of 125 days (95% CI 70-179 days). Both the pulmonary and pediatric pulmonary departments of this CF center had better maintained consistent use rates than all other departments (Figure S3 in Multimedia Appendix 1).

### Impact of Increased Pulmonary Symptoms on Home Spirometry Use Frequency

A total of 381 periods with increased symptoms and 1340 periods with few symptoms were compared. There was a small, but significant difference between the mean number of home spirometry tests performed during periods of increased symptoms (mean=1.33) and those with few symptoms (mean=0.88; ΔMean=0.45, t_497.278_=–4,197; *P*<.001). In the sensitivity analysis, periods of increased and few symptoms could be compared for 76 users. Dependent *t* test showed no significant difference between the number of home spirometry tests performed between these periods (ΔMean=0.37, t_75_=–1.843; *P*=.07). Of the users in the sensitivity analysis, 14 (18.4%) measured only during periods of increased symptoms, 5 (6.6%) only during periods with few symptoms, and 44 (57.9%) measured their lung function in both periods. A total of 13 (17.1%) of all users did not measure their lung function in both periods. In an exploratory analysis, the FEV1 during the first period with increased symptoms after 100 days of use was compared with the nearest period with few symptoms for 44 users with a Wilcoxon signed rank test. The FEV1 was significantly lower during the period with increased symptoms (median=1.75, IQR 1.41-2.59) compared with the period with low symptoms (median=1.97, IQR 1.52-2.89; ΔMedian=0.22; *Z*=–3.46; *P*<.001).

### Influence of Elexacaftor/Tezacaftor/Ivacaftor Treatment Initiation on Use Frequency

**Figure 2:**
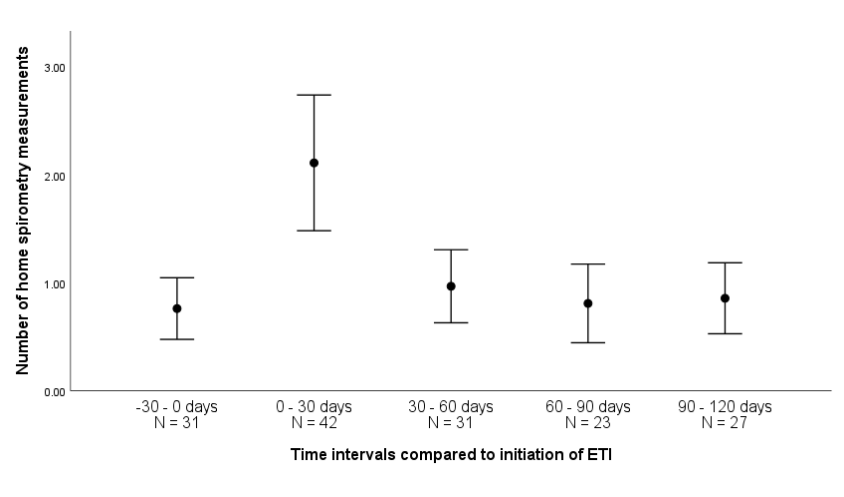
Mean number and 95% CI values of home spirometry measurements per user in the month before and months after initiation of Elexacaftor/Tezacaftor/Ivacaftor treatment. N indicates the number of users who measured their lung function at least once during the intervals.

A total of 63 out of 81 (78%) people with CF for whom additional data were available started Elexacaftor/Tezacaftor/Ivacaftor treatment during the observation period. More users measured their lung function at home within 30 days after initiation of Elexacaftor/Tezacaftor/Ivacaftor (N=42/63, 67%) compared with the other time intervals (Figure 2). The mean number of home spirometry measurements for the consecutive time intervals were 0.76, 2.11, 0.97, 0.81, and 0.86, respectively. Repeated measures ANOVA with Greenhouse-Geyser correction showed a significant effect of time in relation to Elexacaftor/Tezacaftor/Ivacaftor initiation on the number of home spirometry measurements (*F*_2.36,146.26_=13.73, *P*<.001). Tests of within-subjects contrasts showed a significant difference between the month before initiation and 0-30 days after initiation (*F*_1.62_=19, *P*<.001) and between 0-30 days and 30-60 days after initiation (*F*_1.62_=15.37, *P*<.001) but not between 30-60 days and 60-90 days, or 60-90 days and 90-120 days after initiation of Elexacaftor/Tezacaftor/Ivacaftor treatment.

## Discussion

### Principal Findings

This study aimed to examine the use of home spirometry in regular CF care for 2.5 years in 5 Dutch CF centers. Our results showed a fast decrease in both use frequency and the number of users of home spirometry over time. Only a small group of people with CF continued to use home spirometry consistently during the study period. We observed a small but significant increase in the absolute number of home spirometry measurements during periods of increased pulmonary symptoms on a group level as well as shortly after initiation of Elexacaftor/Tezacaftor/Ivacaftor treatment. Moreover, during increased symptoms, up to a fifth of users measured their lung function at home whereas they did not during periods of few pulmonary symptoms.

### Comparison With Literature

Low adherence to home spirometry protocols has been identified as a recurring problem in many prospective trials [8-11,13]. Our results show that the frequency of home spirometry monitoring might be even lower in routine care. This discrepancy could partly be attributed to differences in study designs. First, selection bias is inherent to trials as they select highly skilled and motivated patients or patients with few comorbidities for whom preventing a decline in pulmonary function is the highest priority. Second, this was a retrospective study in the time frame of the COVID-19 pandemic and during the introduction of Elexacaftor/Tezacaftor/Ivacaftor treatment in the Netherlands. These enormous changes in CF care and the lack of a home spirometry protocol might have limited the uptake of home spirometry in our study.

In previous work, we identified multiple incentives (eg, changes in the physical condition and positive psychosocial effects) and disincentives (eg, technical difficulties and lack of perceived need) for regular home monitoring of lung function [7]. Regular use of home spirometry requires patients to allocate time and effort to perform the measurements and this increases the already high treatment burden of people with CF [14-16]. When there are no clear benefits from home spirometry or reductions in overall treatment burden, people with CF might have little motivation to keep measuring their lung function at home regularly [7]. This is emphasized by our findings that an increase in pulmonary symptoms and initiation of Elexacaftor/Tezacaftor/Ivacaftor temporarily increased both the number of users and the number of home spirometry tests. Increases in symptoms and new modulator therapies provide clear incentives and a perceived necessity for people with CF to measure their lung function, but during stable periods these incentives may be less evident.

A previous study showed prolonged uptake in home spirometry in children compared with adults, but the authors also reported that increased supervision of health care professionals in pediatric care might have attributed to this difference [11]. In our study, younger people with CF had just slightly better uptakes of home spirometry than adults. However, we found large differences between CF centers, with much more consistent users in the adult and pediatric departments of one CF center. Interestingly, in this CF center, CF nurses cared for both the adult and pediatric populations. These CF nurses had created a clear strategy for the organization of remote care with policies for the initiation, follow-up, and administration of remote monitoring [7]. This strategy was likely used for both the adult and pediatric populations, whereas similar strategies were not necessarily present in other centers due to the turbulent period of the COVID-19 pandemic [7]. We already know that CF nurses play an important role in the successful implementation of home monitoring interventions, and these findings suggest that differences in uptake might not be due to the age of patients but rather due to the differences in the organization of care and policies regarding home spirometry across CF centers and departments [7]. This strengthens the conclusion that clear agreements within departments and with patients as well as clear policies for the initiation and follow-up of home spirometry may enhance its sustainability.

### Strength and Limitations

To our knowledge, this is the first long-term study into the uptake of home spirometry in a real-world clinical setting. Moreover, our study benefited from the large number of participants, the multicenter setting, and the long follow-up time that increases generalizability to other settings. As stated earlier, the time of initiation of home spirometry was turbulent due to the COVID-19 pandemic and the introduction of Elexacaftor/Tezacaftor/Ivacaftor treatment. This may have affected the implementation of home spirometry and therefore use frequency. Moreover, CF departments were provided with spirometers and access to the RMP but were allowed to implement home spirometry according to their preferences. The uptake of home spirometry differed between CF departments and this may limit our overall findings. But these differences between centers also allowed us to hypothesize about the underlying causes of varying uptake such as the availability of motivated CF nurses and clear policies. Finally, because this study used anonymized data, we were unable to study the frequency of home spirometry within individual contexts such as treatment, health care consumption, socioeconomic background, and other important determinants for digital health use. In addition, due to the use of anonymized data, data on Elexacaftor/Tezacaftor/Ivacaftor treatment use was only available for a small number of users.

### Future Directions

The role of home spirometry in the field of CF and other pulmonary diseases is becoming increasingly evident. However, to ensure that home spirometry does not become an inefficient and underused tool, it is imperative that we regard it as a means to an end instead of a goal on its own [17]. Goals should be tailored to individual needs and contexts, and the intensity of home spirometry monitoring should be adjusted accordingly. Centers should create clear policies for the organization of remote care that address how to set, initiate, follow up, and evaluate these goals on an individual basis to ensure successful implementation.

We previously identified four “Sensible Strategies for Remote Monitoring” as a set of predefined goals that can be adopted in CF care [7]. In our study, CF centers mostly seemed to adopt symptom-driven strategies where home spirometry was used to quantify symptoms when needed rather than at regular intervals. However, the positive attitude from our questionnaire results implies that it is not just the frequency of use that creates the benefits [7]. Nevertheless, some people with CF might benefit from regular home monitoring within patient-driven strategies that address individual needs, but a widespread application seems limited.

There is an increasing interest in prediction-driven strategies that use regular home spirometry to predict a decline in pulmonary function or other clinical outcomes [9,10,18]. However, these strategies only work when people with CF monitor themselves frequently over extended periods of time, also during periods of stability. Our results show these strategies are likely not feasible for the long term or the majority of users. To mitigate this, studies will have to devise solutions to solve the problem of low adherence many of these studies face. They will have to devise solutions and address the lack of perceived necessity for people with CF to measure their lung function during periods of stability. This could include limiting their studies to people with CF who deteriorate often or reducing treatment burdens elsewhere to ensure that home spirometry is not just an additional burden (eg, care-driven strategies that replace regular care with remote monitoring) [15,19].

Hence, there should be a stronger emphasis on whether home spirometry can support individual patients and what intensity of home monitoring best fits individual goals. The frequency of home spirometry on its own is meaningless when not coupled with clear strategies. This approach will not only prevent the introduction of new “monitoring-adherence discussions” in our clinics, but it will also ensure that remote monitoring remains value-driven.

### Conclusion

Consistent uptake of home spirometry in regular long-term multicenter CF care is low but appears to increase around periods of changing symptoms. A clear strategy for the organization of remote care seems to stimulate long-term uptake of home spirometry. Nevertheless, frequent and regular home spirometry monitoring might not be feasible for all patients and all the time. Instead, home spirometry and its intensity are not a goal but should be used as a tool to reach individual patient goals within local contexts.

## Appendix

Multimedia Appendix 1 Kaplan-Meier Plots for the consistent use of home spirometry using strict criteria.

## Abbreviations

CF: cystic fibrosis
CFTR: CF transmembrane conductance regulator
FEV1: forced expiratory volume in one second
RMP: remote monitoring platform
TTN: time-to-next lung function measurement

## Data Availability

Anonymized data from this study will be made available upon reasonable request.
