# Supplementary material for: The Long-Term Uptake of Home Spirometry in Regular Cystic Fibrosis Care: Retrospective Multicenter Observational Study
Source: J Med Internet Res. 2025 Jan 9;27:e60689. doi: 10.2196/60689 (PMC11757947; doi:10.2196/60689)
Supplement: Multimedia Appendix 1 [file jmir_v27i1e60689_app1.docx]

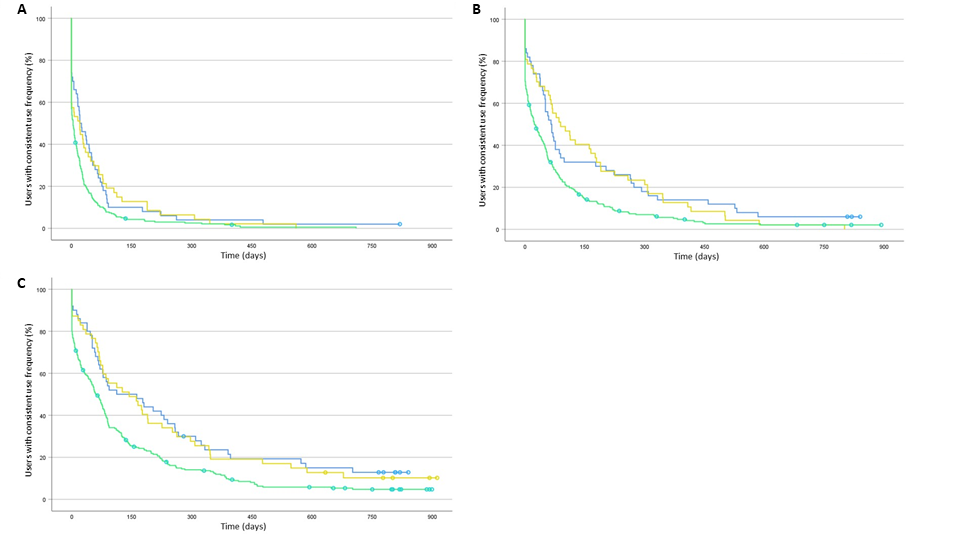

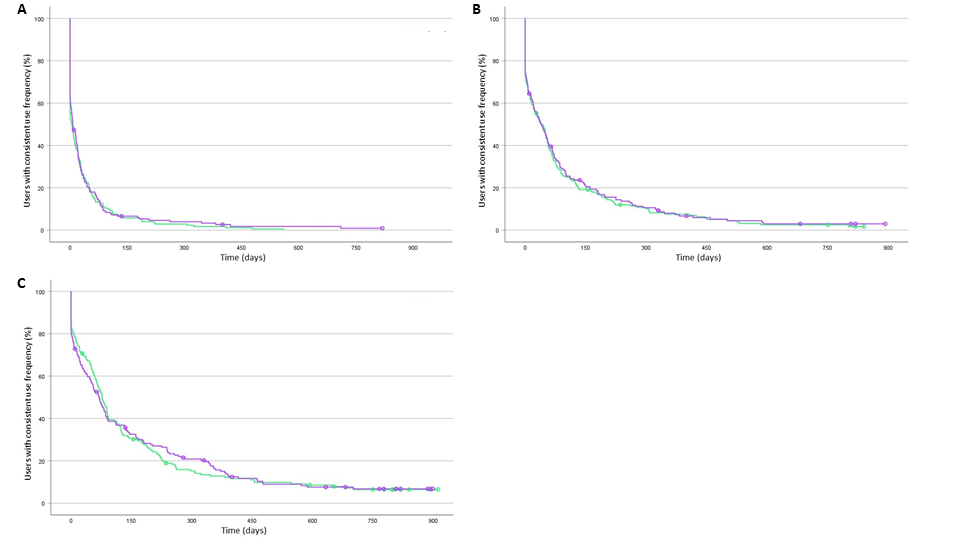


***figure 2****. Kaplan-Meier Plots for the consistent use of home spirometry using strict criteria grouped by sex. Green: male; Purple: female. Panel A: 31 day interval; Panel B: 62 day interval; Panel C: 93 day interval.*

***figure 1****. Kaplan-Meier Plots for the consistent use of home spirometry using strict criteria grouped by age categories. Blue: 6-12 years; Yellow: 12-18 years; Green: >18 years. Panel A: 31 day interval; Panel B: 62 day interval; Panel C: 93 day interval.*

**
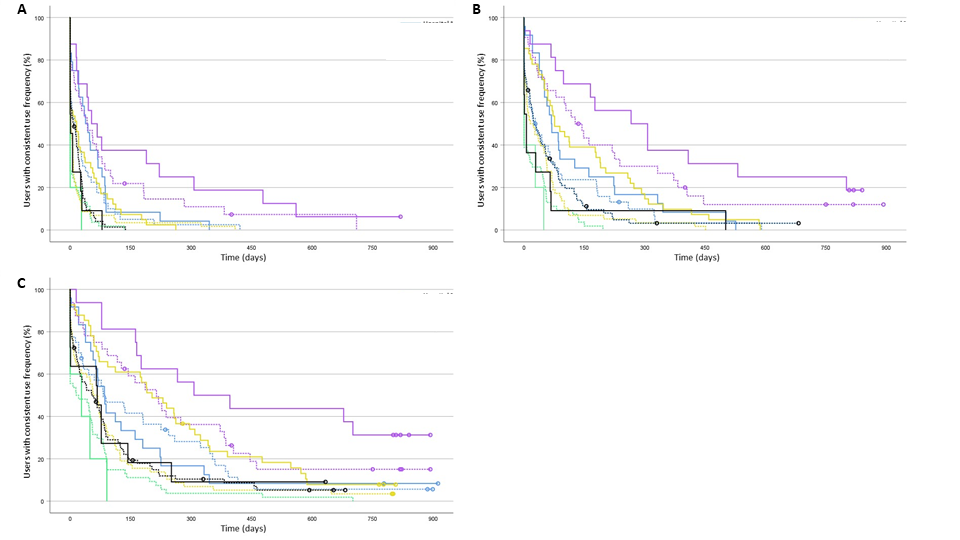

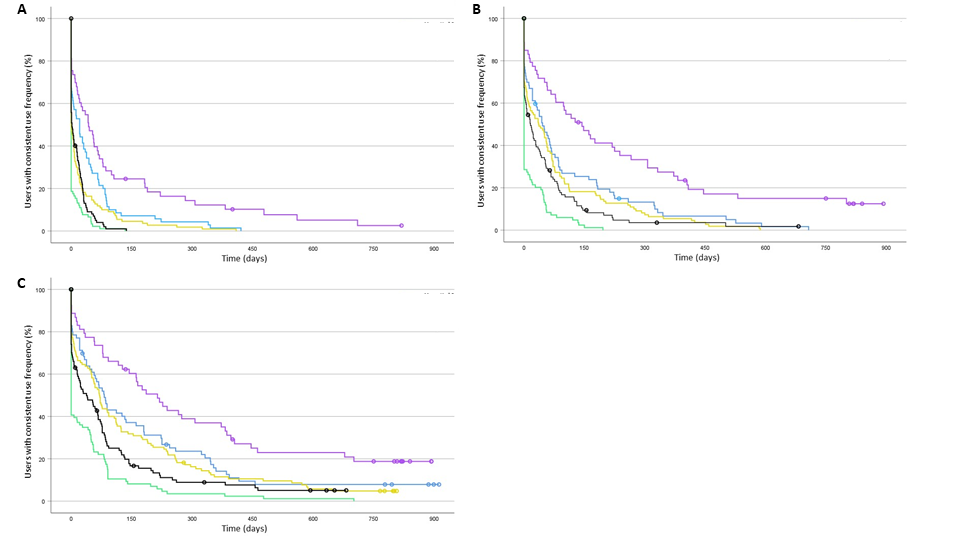
**

***figure 3****. Kaplan-Meier Plots for the consistent use of home spirometry using strict criteria grouped by CF center. Blue: hospital 1; Purple: hospital 2; Green: hospital 3; Yellow: hospital 4: Black: hospital 5. Panel A: 31 day interval; Panel B: 62 day interval; Panel C: 93 day interval.*

***figure 4****. Kaplan-Meier Plots for the consistent use of home spirometry using strict criteria grouped by age categories*treating CF center. Blue: hospital 1; Purple: hospital 2; Green: hospital 3; Yellow: hospital 4: Black: hospital 5. Dashed lines: >18 years; Solid lines: <18 years. Panel A: 31 day interval; Panel B: 62 day interval; Panel C: 93 day interval.*
